# The Relationship Between Cognitive Dysfunction and Symptom Dimensions Across Schizophrenia, Bipolar Disorder, and Major Depressive Disorder

**DOI:** 10.3389/fpsyt.2019.00253

**Published:** 2019-04-26

**Authors:** Yue Zhu, Fay Y. Womer, Haixia Leng, Miao Chang, Zhiyang Yin, Yange Wei, Qian Zhou, Shinan Fu, Xin Deng, Jing Lv, Yanzhuo Song, Yinzhu Ma, Xinyu Sun, Jing Bao, Shengnan Wei, Xiaowei Jiang, Shuping Tan, Yanqing Tang, Fei Wang

**Affiliations:** ^1^Department of Psychiatry, The First Affiliated Hospital of China Medical University, Shenyang, China; ^2^Brain Function Research Section, The First Affiliated Hospital of China Medical University, Shenyang, China; ^3^Department of Psychiatry, Washington University School of Medicine, St Louis, MO, United States; ^4^Department of Radiology, The First Affiliated Hospital of China Medical University, Shenyang, China; ^5^Shanghai Mental Health Center, Shanghai, China; ^6^Center for Psychiatric Research, Beijing Huilongguan Hospital, Beijing, China; ^7^Department of Gerontology, The First Affiliated Hospital of China Medical University, Shenyang, China

**Keywords:** schizophrenia, bipolar disorder, major depressive disorder, symptom, cognitive function

## Abstract

**Background:** Cognitive dysfunction is considered a core feature among schizophrenia (SZ), bipolar disorder (BD), and major depressive disorder (MDD). Despite abundant literature comparing cognitive dysfunction among these disorders, the relationship between cognitive dysfunction and symptom dimensions remains unclear. The study aims are a) to identify the factor structure of the BPRS-18 and b) to examine the relationship between symptom domains and cognitive function across SZ, BD, and MDD.

**Methods:** A total of 716 participants [262 with SZ, 104 with BD, 101 with MDD, and 249 healthy controls (HC)] were included in the study. One hundred eighty participants (59 with SZ, 23 with BD, 24 with MDD, and 74 HC) completed the MATRICS Consensus Cognitive Battery (MCCB), and 507 participants (85 with SZ, 89 with BD, 90 with MDD, and 243 HC) completed the Wisconsin Card Sorting Test (WCST). All patients completed the Brief Psychiatric Rating Scale (BPRS).

**Results:** We identified five BPRS exploratory factor analysis (EFA) factors (“affective symptoms,” “psychosis,” “negative/disorganized symptoms,” “activation,” and “noncooperation”) and found cognitive dysfunction in all of the participant groups with psychiatric disorders. Negative/disorganized symptoms were the most strongly associated with cognitive dysfunctions across SZ, BD, and MDD.

**Conclusions:** Our findings suggest that cognitive dysfunction severity relates to the negative/disorganized symptom domain across SZ, BD, and MDD, and negative/disorganized symptoms may be an important target for effective cognitive remediation in SZ, BD, and MDD.

## Introduction

Schizophrenia (SZ), bipolar disorder (BD), and major depressive disorder (MDD) have long been viewed as distinct disorders based on differing clinical presentations (1); however, there is substantial evidence suggesting that these disorders share pathophysiological and clinical manifestations (2, 3). Moreover, studies have shown co-aggregation of the three disorders in risk genes (4, 5), high familial risk (6), shared neurobiological and neuropsychological features (7), and overlapping syndromes that challenge existing classification criteria (8). Consequently, it has been proposed that SZ, BD, and MDD lie along a continuum of neuropsychiatric illness, rather than represent three separate disorders (3, 9).

Cognitive dysfunction is a core feature across SZ, BD, and MDD (10–12); however, studies of cognition across these diagnoses have yielded mixed results (13). Increasingly, studies have noted similar cognitive patterns and profiles in SZ, BD, and MDD (14–16). To further investigate this theory, cognitive function appears to be a relatively stable intermediate phenotype that may provide insight into the potential link between SZ, BD, and MDD (17, 18). This is further supported by extensive literature implicating a continuum of cognitive dysfunction severity based on severity of neuropsychiatric illness (15, 19, 20). Interestingly, continuum models have also been proposed for psychosis, reflecting shared dimensions of psychopathology across SZ and mood disorders (10, 21).

Studies have examined the relationship between cognitive deficits and symptom dimensions in psychiatric disorders; however, we are not aware of previous studies that examined this relationship across SZ, BD, and MDD. Further, prior studies have focused on the primary symptoms that distinguish SZ, BD, and MDD from each other, limiting direct comparison of psychopathology across SZ, BD, and MDD (22, 23).

The Brief Psychiatric Rating Scale (BPRS) is a very useful measure for psychopathology dimensions across SZ, BD, and MDD. It covers a broad range of symptom domains with efficient and valid assessment of symptom severity (24). While the BPRS is generally used to assess SZ and other psychotic disorders (25, 26), it can also be used to analyze the factor structure in mood disorders (27). Exploratory factor analysis (EFA) identifies the underlying structure of a large variable set such as the BPRS. The structure and associated factors from EFA likely reflect physiological and pathophysiological mechanisms (28). Therefore, using the EFA of BPRS across SZ, BD, and MDD within the context of the same study would present patients their own psychopathological characters, and provide a novel way to help us better understand complex psychiatric disorders better than categorical approaches alone.

The study aims are a) to identify the factor structure of the BPRS-18 and b) to examine the relationship between symptom domains and cognitive function across SZ, BD, and MDD. We hypothesized that cognitive dysfunction are present across SZ, BD, and MDD, relative to healthy controls, and that negative/disorganized symptoms correlate with cognitive dysfunction severity.

## Materials and Methods

### Participants

A total of 716 participants were included in the study: 262 with SZ, 104 with BD, 101 with MDD, and 249 healthy controls (HCs). Patients were recruited from inpatient and outpatient services in the Department of Psychiatry at the First Affiliated Hospital, China Medical University and Shenyang Mental Health Center. HC participants were recruited from the local community of Shenyang using advertisements.

All participants were aged between 18 and 60 years old. Participants with SZ, BD, or MDD were diagnosed according to Diagnostic and Statistical Manual of Mental Disorders-IV-Text Revision (DSM-IV-TR) standards, and the diagnoses were confirmed by two trained psychiatrists using the Structured Clinical Interview for DSM-IV Axis I disorders (SCID-I). HC participants did not have current or life Axis I disorders and any first-degree relatives with a history of Axis I disorders. Participants were excluded for the following: concomitant major medical disorder, neurological disease or head injury with loss of consciousness, and/or substance/alcohol abuse or dependence.

This study was approved by the Medical Research Ethics Committee of the China Medical University in accordance with the Declaration of Helsinki. All participants gave written informed consent, or the parents or legal guardian for participants <18 years, after receiving a detailed description of the study.

### Measures

All patients completed the BPRS-18 (29) to assess current psychopathology.

Cognitive function was assessed in a subset of patients and HC using the following: 1) MATRICS Consensus Cognitive Battery (MCCB) (30) [the battery is composed of 10 subtests across seven domains, including a) Speed of Processing: Trail Making Test-Part A (TMT-A), Symbol Coding, and Category Fluency; b) Working Memory (WM): Visual WM (Spatial Span) and Verbal WM (Letter-Number Span); c) Verbal Learning: Hopkins Verbal Learning Test-Revised (HVLT-R); d) Visual Learning: Brief Visuospatial Memory Test-Revised (BVMT-R); e) Reasoning and Problem Solving: Mazes; f) Attention: Continuous Performance Test-Identical Pairs version (CPT-IP), which measures the mean d’ score among the three conditions; and (g) Social Cognition: The Mayer–Salovey–Caruso Emotional Intelligence Test (MSCEIT); 59 SZ, 23 BD, 24 MDD, and 74 HC subjects completed the MCCB] and 2) Wisconsin Card Sorting Test (WCST) (31). A computerized version of the WCST was given. The WCST evaluates executive function and provides subscores as follows: correct responses (CR), categories completed (CC), total errors (TE), perseverative errors (PE), and non-perseverative errors (NPE). Eighty-five SZ, 89 BD, 90 MDD, and 243 HC subjects completed the WCST. There were 137 SZ, 11 BD, and 11 MDD without cognitive test. This study included these patients used to assess psychopathology.

For each participant, clinical and cognitive assessments were completed within 1 week.

### Statistical Analysis

ANOVAs (analyses of variance) or chi-square tests were used to examine participants’ demographic characteristics (age and sex) and clinical characteristics (duration of illness, age of first episode, first episode, and medication status) accordingly.

EFA was performed for BPRS scores in the patient groups only. Orthogonal rotation was accomplished using the Varimax method. The number of factors retained was determined based on eigenvalues >1, and the numbers were confirmed by the screen plot cutoff point. In order to accurately interpret the factor structure and contents, we assumed that there was only a loading of more than 0.30 for any given variable to be significant (32). ANOVA and LSD’s *post hoc* analysis were used to compare the factor structure scores and total score of BPRS among patient groups (SZ, BD, and MDD).

Cognitive measures (MCCB and WCST) were analyzed using ANCOVA (analyses of covariance) and LSD’s *post hoc* analyses, with sex, age, and years of education as covariates. For the MCCB, raw scores were used for each subtest.

Partial correlation was used to determine the relationship between BPRS EFA factor scores and cognitive measures in patient group as a whole and separately, after controlling for sex and age. False discovery rate correction was used for multiple comparisons.

Based on previous correlation analyses, we then used multiple regression analyses to examine the effects of clinical symptom scores on cognitive outcomes after accounting for the above demographic and clinical characteristics (diagnosis, duration of illness, age of first episode, first episode, and medication).

Significance was set at P < 0.05 (two-tailed) for all tests. All analyses were performed using SPSS 22.0.

## Results

### Demographic Data of Participants

There were significant differences in age and sex among the SZ, BD, MDD, and HC groups. Patient groups also significantly differed in duration of illness, age of first episode, first episode status, and medication status (**Table 1**).

**Table 1:** Demographic and clinical characteristics of schizophrenia (SZ), bipolar disorder (BD), major depressive disorder (MDD), and healthy controls (HC).

	SZ N = 262	BD N = 104	MDD N = 101	HC N = 249	F/χ^2^ values	P values
Demographic characteristics						
Age	34.40 (11.09)	30.06 (9.88)	32.37 (9.26)	33.52 (11.69)	2.871^a^	0.036
Sex, Female	152 (58.0%)	64 (61.5%)	82 (81.2%)	154 (61.8%)	18.652^a^	0.001
Years of Education	11.74 (3.21)	13.29 (3.21)	13.11 (3.05)	14.43 (3.56)	27.483^a^	<0.001
Clinical characteristics						
Duration of Illness, Months^c^	71.98 (86.19)	59.02 (66.54)	23.98 (45.45)	–	12.262^b^	<0.001
Age of First Episode^c^	26.21 (8.46)	25.34 (9.23)	29.43 (10.10)	–	5.805^b^	0.03
First Episode, Yes^c^	110 (43.3%)	39 (38.6%)	78 (78.0%)	–	41.147^b^	<0.001
Medication, Yes^c^	209 (80.1%)	75 (72.1%)	59 (58.4%)	–	17.739^b^	<0.001

Data are n (%) or mean (SD).

HAMD, Hamilton Depression Scale; HAMA, Hamilton Anxiety Scale; YMRS, Young Rating Scale.

^a^Is the examination among SZ, BD, MDD, and HC; ^b^is the examination among SZ, BD, and MDD; ^c^is information that was missing for some participants.

The age and sex were not matched among SZ, BD, MDD, and HC. Clinical characteristics were significant differences in SZ, BD, and MDD.

### Exploratory Factor Analysis

The sample size (n = 467) of our study was above the minimum recommended for EFA (n > 150). Further, the Kaiser–Meyer–Olkin value of our study was 0.783, which exceeds the minimum recommended value (33). Importantly, Bartlett’s test for sphericity was significant [X^2^ (153) = 2222.292, P < 0.001]. As such, all indicators support the suitability of the study’s data for EFA.

There were five eigenvalues greater than 1.0. Furthermore, there was a clear change in the slope of the eigenvalue plot after the fifth factor, which determined the number of factors we computed. After Varimax rotation, we identified five interpretable and clinically relevant factors that captured 58.41% of the rotated variance. All of the BPRS-18 items were included in the EFA. **Table 2** lists the five resulting symptom factor structures and their item loadings with absolute values greater than 0.30.

**Table 2:** The five-factor solution for Brief Psychiatric Rating Scale (BPRS).

Dimension	Eigenvalue	Variance (%)^a^	Item	Loading
Affective symptoms	4.03	22.41	Depression	0.84
			Anxiety	0.82
			Guilt	0.67
			Tension	0.67
			Somatic Concern	0.60
Psychosis	2.46	13.65	Unusual Thought Content	0.78
			Suspiciousness	0.77
			Hostility	0.67
			Hallucinations	0.66
Negative/disorganized symptoms	1.60	8.91	Emotional Withdrawal	0.72
			Blunted Affect	0.69
			Motor Retardation	0.62
			Conceptual Disorganization	0.66
			Disorientation	0.57
Activation	1.31	7.26	Excitement	0.77
			Mannerisms and Posturing	0.70
			Grandiosity	0.41
Noncooperation	1.11	6.18	Uncooperativeness	0.73

a Is the cumulative variance, which is 58.41%.

Affective symptoms were composed of Depression, Anxiety, Guilt, Tension, and Somatic Concern. Psychosis was composed of Unusual Thought Content, Suspiciousness, Hostility, and Hallucinations. Negative/Disorganized Symptoms were composed of Emotional Withdrawal, Blunted Affect, Motor Retardation, Conceptual Disorganization, and Disorientation. Activation was composed of Excitement, Mannerisms and Posturing, and Grandiosity. Noncooperation included only one Uncooperativeness item.

The BPRS EFA factors are as follows: 1) “affective symptoms” (includes depression, anxiety, guilt, tension, and somatic concerns), 2) “psychosis” (includes unusual thought content, suspiciousness, hostility, and hallucinations), 3) “negative/disorganized symptoms” (includes emotional withdrawal, blunted affect, motor retardation, conceptual disorganization, and disorientation), 4) “activation” (includes excitement, mannerisms and posturing, and grandiosity), and 5) “noncooperation” (consists only of uncooperativeness).

**Figure 1:**
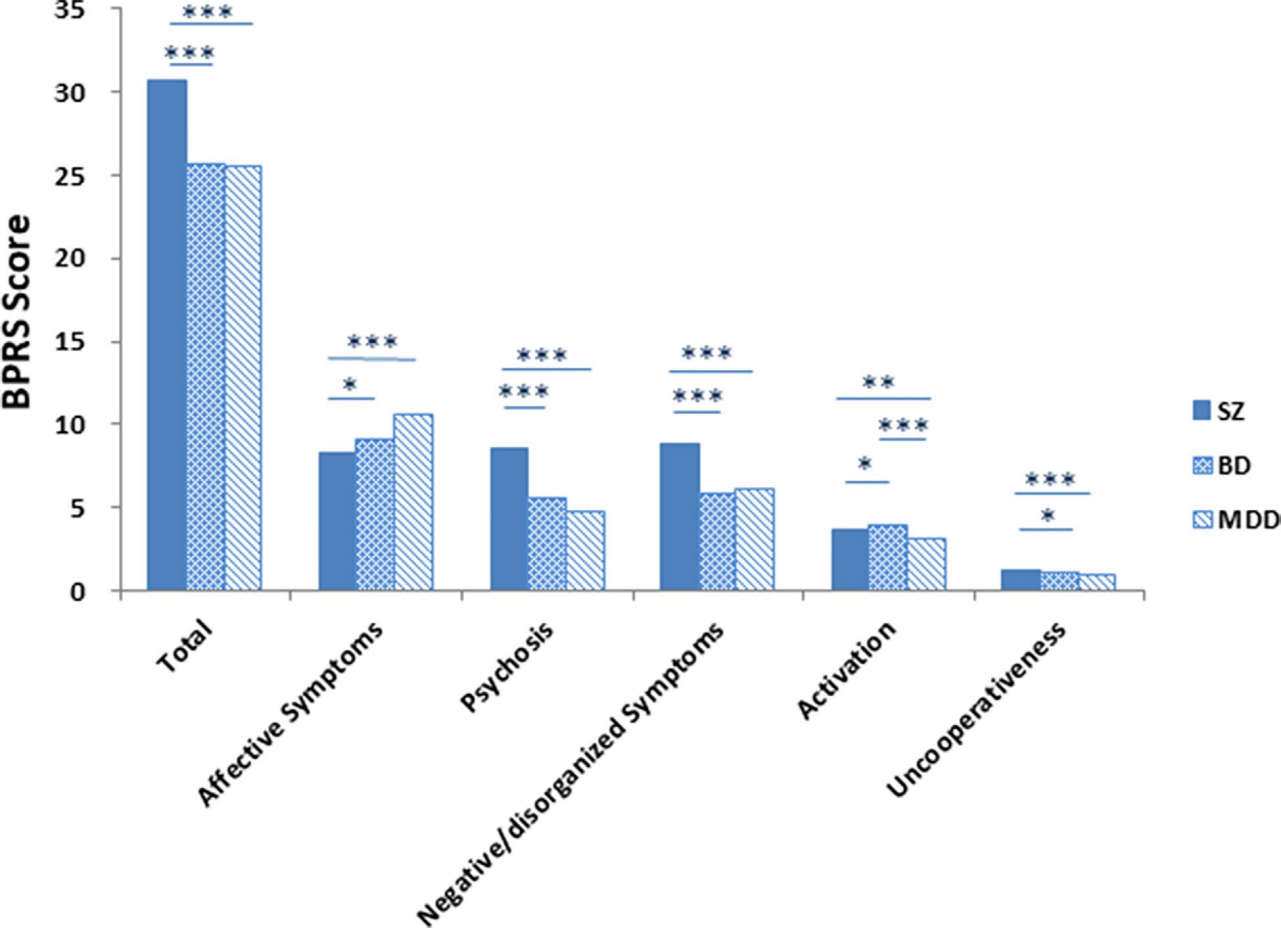
The Brief Psychiatric Rating Scale (BPRS) total scores and factor scores by diagnosis. Note: *P < 0.05, **P < 0.01, ***P < 0.001. Patient groups differed on BPRS scores. Bipolar disorder (BD) and major depressive disorder (MDD) groups differed from the schizophrenia (SZ) in “total score,” “affective symptoms,” “psychosis,” “negative/disorganized symptoms,” and “uncooperativeness” dimensions scores but did not differ from each other. For “activation,” SZ, BD, and MDD differed from each other.

Total scores and factor scores of BPRS were significantly different among the patient groups. *Post hoc* analysis revealed that the BD and MDD groups significantly differed from the SZ group in BPRS total score and EFA factor scores of “affective symptoms,” “psychosis,” “negative/disorganized symptoms,” and “noncooperation.” There were no significant differences between BD and MDD groups. There were significant differences in “activation” scores between SZ, BD, and MDD (**Figure 1**).

### Cognitive Measures

Compared to the HC group, the SZ, BD, and MDD groups were significantly impaired in Maze but did not differ from each other. Compared to the HC group, the SZ and BD groups were significantly impaired in TMT-A, Symbol Coding, Spatial Span, Letter-Number Span, BVMT-R, and CPT-IP, and the SZ and MDD groups were significantly impaired in HVLT-R. In Symbol Coding, Spatial Span, HVLT-R, and CPT-IP, BD and MDD differed from SZ but did not differ from each other. In TMT-A, Letter-Number Span, and BVMT-R, SZ differed from MDD. In WCST, comparisons of the HC, SZ, BD, and MDD groups yielded significant differences in CR, CC, TE, and NPE, and SZ and MDD had significant differences in PE. BD and MDD differed from the SZ in CC but did not differ from each other. In CR, TE, and NPE, there were differences between SZ and BD. There was no significant difference between BD and MDD in MCCB and WCST (**Table 3**).

**Table 3:** Cognitive performance of SZ, BD, MDD, and HC.

	SZ	BD	MDD	HC	ANOVA		
					F	P	*Post hoc*
MCCB							
N	59	23	24	74			
TMT-A	89.22 (72.29)	61.00 (45.18)	46.44 (30.26)	41.47 (24.22)	10.723	<0.001	SZ > MDD, HC; BD > HC
Symbol Coding	38.87 (15.91)	53.87 (14.02)	58.96 (11.98)	59.55 (16.00)	28.946	<0.001	SZ < BD < HC; SZ < MDD
Category Fluency	18.25 (7.04)	20.39 (6.67)	20.50 (5.22)	21.649 (5.96)	2.301	0.079	
Spatial Span	11.37 (5.29)	14.61 (6.52)	16.75 (4.50)	17.22 (4.97)	15.862	<0.001	SZ < BD < HC; SZ < MDD
Letter-Number Span	17.25 (5.99)	20.39 (6.79)	22.64 (3.76)	22.85 (4.59)	11.784	<0.001	SZ < MDD, HC; BD < HC
HVLT-R	19.66 (7.01)	26.46 (7.83)	26.20 (4.19)	27.57 (5.00)	21.704	<0.001	SZ < MDD < HC; SZ < BD
BVMT-R	17.15 (8.90)	23.86 (8.63)	24.83 (6.04)	25.97 (7.92)	16.033	<0.001	SZ < BD < HC; SZ < MDD
Mazes	8.95 (6.37)	13.14 (6.82)	12.71 (6.09)	15.18 (6.56)	11.832	<0.001	SZ, BD, MDD < HC
CPT-IP	−0.43 (0.95)	0.52 (0.76)	0.74 (0.50)	0.85 (0.54)	37.723	<0.001	SZ < BD < HC; SZ < MDD
MSCEIT	9.02 (2.39)	8.98 (1.70)	8.31 (2.47)	8.88 (1.71)	2.232	0.086	
WCST							
N	85	89	90	243			
CR	19.53 (12.47)	25.12 (12.17)	21.84 (11.25)	28.34 (12.72)	14.444	<0.001	SZ < BD < HC; MDD < HC
CC	1.94 (2.17)	3.01 (2.13)	2.61 (1.94)	3.55 (2.25)	14.144	<0.001	SZ < BD, MDD < HC
TE	28.54 (12.52)	22.54 (12.19)	26.22 (11.23)	19.74 (12.80)	14.353	<0.001	SZ > BD > HC; MDD > HC
PE	11.81 (10.98)	9.25 (8.91)	11.10 (9.57)	7.45 (7.96)	6.194	<0.001	SZ > HC; MDD > HC
NPE	16.73 (8.74)	13.63 (7.00)	15.13 (7.24)	12.16 (7.21)	9.714	<0.001	SZ > BD > HC; MDD > HC

Data are mean (SD).

MCCB, The MATRICS Consensus Cognitive Battery; TMT-A, Trail Making Test–Part A; HVLT-R, Hopkins Verbal Learning Test–Revised; BVMT-R, Brief Visuospatial Memory Test–Revised; CPT-IP, Continuous Performance Test–Identical Pairs version; MSCEIT, The Mayer–Salovey–Caruso Emotional Intelligence Test; WCST, The Wisconsin Card Sorting Test; CR, correct responses; CC, categories completed; TE, total errors; PE, perseverative errors; NPE, nonperseverative errors.

### Correlations Between Clinical Symptom Dimensions and Cognition

“Negative/disorganized symptoms” was significantly correlated with neurocognitive function, in most subtests of MCCB (TMT-A, Symbol Coding, Spatial Span, Letter-Number Span, HVLT-R, Mazes, and CPT-IP) and WCST (CR, CC, TE, and NPE) across SZ, BD, and MDD, after false discovery rate correction. Correlations between clinical symptom dimensions and cognition in individual patients groups can be found in **Supplementary Materials** (**Supplementary Table 1**).

Multiple regression analyses showed that “negative/disorganized symptoms” predicted scores for HVLT-R (β = −0.402, t = −2.039, P = 0.045), Mazes (β = −0.382, t = −2.084, P = 0.041), CPT-IP (β = −0.087, t = −3.347, P = 0.001), CR (β = −0.999, t = −4.272, P < 0.001), CC (β = −0.157, t = −3.865, P < 0.001), TE (β = 1.029, t = 4.39, P < 0.001), PE (β = 0.484, t = 2.34, P = 0.02), and NPE (β = 0.488, t = 3.248, P = 0.001) across all patient groups (**Table 4**).

**Table 4:** Correlations between Clinical Symptom Dimensions and Cognition across SZ, BD and MDD.

	BPRS Total	Affective symptoms	Psychosis	Negative/ Disorganized symptoms	Activation	Noncooperation
MCCB						
TMT-A	0.134(0.199)	0.017(0.867)	0.037(0.724)	0.289(0.005)**	0.032(0.759)	−0.095(0.363)
Symbol Coding	−0.185(0.075)	0.087(0.407)	−0.159(0.127)	−0.281(0.006)**	−0.193(0.063)	−0.059(0.571)
Category Fluency	0.005(0.965)	−0.007(0.943)	0.143(0.170)	−0.201(0.052)	0.084(0.420)	−0.065(0.532)
Spatial Span	−0.129(0.217)	0.099(0.345)	−0.087(0.407)	−0.238(0.021)*	−0.187(0.071)	−0.086(0.410)
Letter Number Span	−0.250(0.015)*	−0.068(0.515)	−0.153(0.142)	−0.289(0.005)**	−0.168(0.105)	−0.020(0.848)
HVLT-R	−0.019(0.854)	0.166(0.109)	0.061(0.562)	−0.256(0.013)*	−0.145(0.163)	−0.032(0.758)
BVMT-R	−0.110(0.290)	0.008(0.939)	−0.052(0.619)	−0.196(0.058)	−0.074(0.478)	−0.009(0.930)
Mazes	−0.153(0.140)	0.017(0.873)	−0.095(0.361)	−0.234(0.023)*	−0.120(0.249)	−0.042(0.685)
CPT-IP	−0.075(0.473)	0.139(0.181)	−0.021(0.841)	−0.273(0.008)**	−0.117(0.263)	−0.073(0.486)
MSCEIT	−0.078(0.454)	−0.147(0.158)	0.018(0.864)	−0.044(0.672)	0.054(0.604)	−0.184(0.076)
WCST						
CR	−0.144(0.024)*	−0.003(0.959)	−0.101(0.114)	−0.282(<0.001)***	−0.049(0.445)	−0.076(0.237)
CC	−0.148(0.020)*	−0.009(0.886)	−0.140(0.028)*	−0.234(<0.001)***	−0.074(0.247)	0.028(0.666)
TE	0.152(0.017)*	0.007(0.908)	0.113(0.077)	0.287(<0.001)***	0.040(0.534)	0.079(0.216)
PE	0.125(0.050)	0.018(0.780)	0.122(0.055)	0.186(0.003)**	0.018(0.783)	0.032(0.614)
NPE	0.074(0.249)	−0.017(0.786)	0.010(0.874)	0.213(0.001)**	0.054(0.398)	0.108(0.091)

Data are presented as r-value (P-value). Bold indicates significance at p < 0.05, after false discovery rate correction.

*P < 0.05, **p < 0.01, ***p < 0.001.

## Discussion

In this study, we identified the factor structure of the BPRS-18 in SZ, BD, and MDD and examined the relationship between BPRS EFA factors and cognitive function across the three disorders. We determined five BPRS EFA factors from EFA: affective symptoms, psychosis, negative/disorganized symptoms, activation, and noncooperation. Cognitive impairment was observed in all patient groups, compared to HC, and significant differences in some cognitive measures were noted between patient groups. “Negative/disorganized symptoms” was positively correlated with several cognitive measures across SZ, BD, and MDD and was a significant predictor of performance for several cognitive tests (attention, verbal learning, problem reasoning and solving, and executive function).

Importantly, our findings support prior evidence that cognitive dysfunction severity positively correlates with certain dimensions of psychopathology, particularly negative/disorganized symptoms (10).

### Dimensions of Psychopathology

The National Institute of Mental Health (NIMH) Research Domain Criteria (RDoC) is an emerging research framework that focuses on the dimensional aspects of neuropsychiatric illness based on neural systems rather than a disorder-specific approach. BPRS appears to be a useful tool for quantifying psychopathology across disorders that can be used easily in research and clinical settings. Prior studies have also examined the factor structure of the BPRS. Velligon et al. identified a four-factor structure (depression/anxiety, psychosis, negative symptoms, and activation) using EFA of the 24-item BPRS across SZ, BD, and MDD (34). Prior EFA of the BPRS-18 identified four factors in depression (citations): EFA factors of apathy, dysphoria, depression, and psychoticism in 163 unipolar depressive patients (35) and of mood disturbance, positive symptoms/apathy, bipolarity, and thought distortion/mannerism in 258 patients with MDD (32). The differences in BPRS-18 factor structure found herein may relate to sample size, a critical variable in EFA, and sample clinical characteristics. A factor analysis study on the BPRS has resulted in a relatively high number of factors - four or five factors (36). In our study, the sample size was 467, and a five-factor solution was determined for the BPRS-18: affective symptoms, psychosis, negative/disorganized symptoms, activation, and noncooperation. The five-factor solution presents patients their own psychopathological characters using the EFA of BPRS in our study instead of previous works.

### Comparisons of Cognitive Outcomes

In our study, cognitive dysfunction appeared most severe in SZ, followed by BD and then MDD, compared to HC. For the MCCB, the SZ group had a wider range of cognitive deficits than BD and MDD. Compared to HC, the BD group had significantly lower scores in six subtests, whereas the MDD group was only lower in two subtests. Intriguingly, Simonsen et al. found that cognitive impairment correlated more with history of psychosis than diagnosis in SZ, schizoaffective disorder (SAD), and BD subjects (37).

Empirical studies and meta-analyses have also shown greater degree of cognitive dysfunction in SZ than in BD and MDD with psychosis (19, 38–40). Other studies have found similar patterns in SZ and affective psychoses, although cognitive dysfunction severity varied across diagnosis (21, 39, 41). Cognitive function is important in functional outcomes in SZ and affective disorders (42, 43). In addition, cognitive deficits often predict similar functional outcomes in affective disorders (44).

Taken together, these findings suggest that cognitive dysfunction may relate more to psychopathology severity rather than the diagnosis itself in SZ, BD, and MDD.

### Correlations Between Psychopathological Dimensions and Cognition

The correlations between clinical symptoms and cognitive functions in SZ and mood disorders are complicated. In our study, “negative/disorganized symptoms” was prominently associated with cognitive dysfunction across SZ, BD, and MDD.

However, “negative/disorganized symptoms” was significantly but weakly correlated with TMT-A, Category Fluency, and Spatial Span in MDD after FDR correction. This may be due to the small sample size of each patient group. Besides, we still could find the tendency of negative/disorganized symptoms playing potential role in associating with cognition. Many prior studies have failed to show an association between positive symptoms (e.g., hallucinations and delusions) and cognitive dysfunction in SZ (45, 46).

Instead, previous studies have more consistently found an association between negative symptoms and cognitive dysfunction in SZ and BD (10), as well as between negative and disorganized symptoms and cognitive dysfunction (45, 47, 48). Some studies have also found negative symptoms and cognitive dysfunction may be separable in SZ and no association between disorganized symptoms and cognitive dysfunction in SZ (49, 50). These inconsistent findings could be attributed to differences in illness duration or stage. Studies examining the relationship between positive and negative symptoms and cognition in MDD are scarce. They have found that affective symptoms were weakly linked to cognitive impairments in SZ and schizoaffective disorders (50, 51).

## Limitations

There were several limitations in this study. Most patients were taking psychotropic medications at the time of study participation. There were significant differences in sex among the participant groups: There were four times more women than men in the MDD group. Psychiatric status (active versus remitted illness) was not accounted for in any of the patient groups. Lastly, there was only a small subset of participants who completed the MCCB. Further work is needed in a larger sample to confirm results reported here.

## Conclusion

Cognitive dysfunction is present across SZ, BD, and MDD, although in varying severity. Across these disorders, negative/disorganized symptoms appear most prominently correlated with cognitive dysfunction than other symptom domains, suggesting that cognitive dysfunction severity is not necessarily based on diagnosis. These findings suggest that negative/disorganized symptoms may be an important target for effective cognitive remediation in SZ, BD, and MDD.

## Ethics Statement

This research was approved by the Medical Research Ethics Committee of the China Medical University and in accordance with the Declaration of Helsinki. All participants gave written informed consent, and the adolescent participants’ parents or legal guardian provided written informed consent after receiving a detailed description of the study.

## Author Contributions

YZ, MC, YT, and FW designed the study. HL, ZY, YW, QZ, SF, XD, JL, YS, YM, XS, JB, SW, and XJ acquired the data. YZ and ST analyzed the data. YZ and FYW wrote the article.

## Conflict of Interest Statement

The authors declare that the research was conducted in the absence of any commercial or financial relationships that could be construed as a potential conflict of interest.

## Supplementary Material

Supplementary Table 1 Correlations between clinical symptom dimensions and cognition across SZ, BD, and MDD.
