# Supplementary material for: The Relationship Between Cognitive Dysfunction and Symptom Dimensions Across Schizophrenia, Bipolar Disorder, and Major Depressive Disorder
Source: Front Psychiatry. 2019 Apr 26;10:253. doi: 10.3389/fpsyt.2019.00253 (PMC6498739; doi:10.3389/fpsyt.2019.00253)
Supplement: Supplementary Table 1 — Correlations between clinical symptom dimensions and cognition across SZ, BD, and MDD. [file Table_1.pdf]

Supplemental Table 1. Correlations between Clinical Symptom Dimensions and Cognition in SZ, BD and MDD

	BPRS Total			Affective symptoms			Psychosis			Negative/ Disorganized symptoms		
	SZ	BD	MDD	SZ	BD	MDD	SZ	BD	MDD	SZ	BD	MDD
MCCB												
TMT-A	0.114(0.419)	0.067(0.780)	0.125(0.579)	0.230(0.100)	-0.252(0.284)	-0.203(0.364)	-0.045(0.754)	0.232(0.326)	0.261(0.240)	0.193(0.171)	0.256(0.275)	**0.659(0.001)**
Symbol Coding	-0.182(0.195)	-0.087(0.715)	-0.121(0.593)	-0.336(0.015)	0.335(0.149)	0.107(0.636)	0.024(0.868)	-0.289(0.216)	-0.177(0.430)	-0.151(0.284)	-0.195(0.411)	-0.473(0.026)
Category Fluency	-0.035(0.807）	0.015(0.951)	-0.329(0.135)	-0.305(0.028)	0.336(0.148)	-0.071(0.754)	0.199(0.158)	-0.216(0.361)	-0.280(0.207)	-0.170(0.229)	-0.077(0.747)	**-0.590(0.004)**
Spatial Span	-0.055(0.697)	0.091(0.702)	-0.463(0.030)	-0.010(0.946)	0.286(0.222)	-0.201(0.370)	0.049(0.732)	0.010(0.967)	-0.084(0.709)	-0.132(0.352)	0.056(0.815)	**-0.737(<0.001)**
Letter-Number Span	-0.299(0.031）	0.123(0.605)	-0.529(0.011)	**-0.460(0.001)**	0.347(0.134)	-0.454(0.034)	-0.146(0.302)	0.157(0.510)	-0.126(0.577)	-0.180(0.201)	-0.153(0.521)	-0.344(0.117)
HVLT-R	0.026(0.857)	0.097(0.683)	-0.149(0.508)	-0.043(0.761)	0.351(0.130)	0.013(0.956)	0.198(0.158)	0.045(0.852)	0.105(0.643)	-0.164(0.245)	0.070(0.770)	-0.458(0.032)
BVMT-R	-0.074(0.060）	0.111(0.640)	-0.200(0.371)	**-0.394(0.004)**	0.366(0.112)	-0.121(0.591)	0.104(0.465)	0.108(0.651)	-0.161(0.474)	-0.115(0.419)	-0.136(0.569)	-0.194(0.386)
Mazes	-0.235(0.094）	-0.046(0.846)	-0.201(0.370)	-0.184(0.192)	0.223(0.345)	-0.007(0.976)	-0.059(0.680)	-0.255(0.277)	-0.209(0.350)	-0.225(0.109)	-0.094(0.695)	-0.423(0.050)
CPT-IP	-0.063(0.655)	-0.108(0.650)	0.252(0.258)	-0.100(0.481)	0.370(0.108)	-0.089(0.694)	0.139(0.324)	-0.376(0.103)	-0.165(0.463)	-0.223(0.112)	-0.229(0.330)	-0.397(0.067)
MSCEIT	-0.077(0.589)	-0.016(0.947)	-0.041(0.858)	-0.226(0.108)	-0.189(0.426)	0.036(0.873)	-0.018(0.901)	0.095(0.691)	0.475(0.025)	0.027(0.847)	0.052(0.828)	-0.376(0.084)
WCST												
CR	-0.134(0.229)	-0.058(0.596)	-0.072(0.506)	-0.020(0.856)	-0.014(0.901)	-0.029(0.790)	-0.012(0.914)	-0.014(0.899)	0.019(0.862)	-0.257(0.019)	-0.108(0.319)	-0.198(0.064)
CC	-0.091(0.414)	-0.027(0.807)	-0.139(0.196)	0.025(0.825)	0.007(0.948)	-0.109(0.310)	-0.057(0.607)	0.008(0.940)	-0.028(0.794)	-0.149(0.179)	-0.098(0.366)	-0.185(0.085)
TE	0.141(0.204)	0.071(0.511)	0.065(0.549)	0.028(0.800)	0.026(0.81)	0.022(0.841)	0.018(0.868)	0.034(0.753)	-0.022(0.839)	0.256(0.019)	0.121(0.262)	0.195(0.068)
PE	0.195(0.078)	0.014(0.894)	0.073(0.498)	0.203(0.065)	-0.084(0.438)	0.007(0.947)	0.076(0.493)	0.101(0.352)	-0.024(0.826)	0.171(0.121)	0.058(0.591)	0.241(0.024)
NPE	-0.029(0.798)	0.079(0.467)	0.001(0.995)	-0.208(0.059)	0.132(0.221)	0.026(0.812)	-0.061(0.585)	-0.108(0.320)	-0.002(0.984)	0.168(0.128)	0.108(0.322)	-0.028(0.798)

Note: Data are presented as r-value (P-value). Bold indicates significance at $p< 0.05$, after false discovery rate correction.

There was no significant association between "Activation" and "Noncooperation" with cognition.
